# Maternal depression during the perinatal period: the role of Sensory Processing Sensitivity and social support and its impact on infants’ negative affect

**DOI:** 10.3389/fpsyg.2025.1551016

**Published:** 2025-03-20

**Authors:** Alessandra Sperati, Ilenia Passaquindici, Melba Emilia Persico, Cinzia Di Matteo, Mirco Fasolo, Francesca Lionetti, Maria Spinelli

**Affiliations:** ^1^Department of Neuroscience, Imaging and Clinical Sciences, University G. d’Annunzio Chieti-Pescara, Chieti, Italy; ^2^Department of Psychology, University G. d’Annunzio Chieti-Pescara, Chieti, Italy; ^3^ASL 02, Lanciano-Vasto-Chieti, Chieti, Italy; ^4^Department of Brain and Behavioral Sciences, University of Pavia, Pavia, Italy

**Keywords:** maternal depression, Sensory Processing Sensitivity (SPS), partner support, negative affect trait, infants, perinatal period, depression trajectories

## Abstract

**Background:**

The perinatal period is marked by significant physiological and psychological changes, making it a challenging time for many women. While some women are more vulnerable to depression during this period, research on perinatal depression trajectories and contributing factors remains mixed. This longitudinal study investigated how maternal depression changes during the perinatal period in a non-clinical sample, exploring the roles of individual factors, such as Sensory Processing Sensitivity (SPS), and contextual factors, such as global partner support. Based on the prenatal programming hypothesis, we also examined the role of prenatal depression on infants’ negative affect temperament as an early marker of emotional adjustment.

**Method:**

Eighty-eight mothers (*M* = 35.03 years, *SD* = 4.92) completed online questionnaires during pregnancy, at 3, 6, and 9 months post-partum. Depression was assessed using the Edinburgh Postnatal Depression Scale, partner support was measured with an ad-hoc scale. SPS was reported during pregnancy using the Highly Sensitive Person Scale. At 3 months post-partum, infants’ negative affect (*M* = 3.07 months, *SD* = 0.26) was measured using the Infant Behavior Questionnaire. Latent growth analyses and multivariate regression models were used to analyze the data.

**Results:**

Depression showed a significant linear decrease, with higher symptoms between pregnancy and 3 months postpartum, although overall levels were low and consistent with non-clinical populations. High SPS predicted greater depressive symptoms across all time points. Pregnancy partner support was associated with lower depressive symptoms during pregnancy (*β* = −0.42, *p* < 0.01) and at 6 months postpartum (*β* = −0.32, *p* = 0.03). Prenatal depression significantly predicted infants’ negative affect (*β* = 0.34, *p* = 0.03), particularly the fear temperament subscale (*β* = 0.46, *p* = 0.001), accounting for 22% of its variance.

**Discussion:**

The findings clarify that a decline in mood is common in non-clinical populations during the perinatal period, with mothers high in sensitivity and mothers with lower partner support being more vulnerable to experience negative feelings. Moreover, prenatal maternal depression acts as a prenatal stressor, increasing infants’ reactivity to stimuli, as reflected in heightened fear. Implications for tailored parenting programs are discussed.

## Introduction

The transition to motherhood is a crucial period marked by significant physiological, relational, and psychological changes, making the perinatal phase potentially challenging. Women may experience these physical and emotional demands differently, with approximately 10–25% of them being more vulnerable to depression from pregnancy up to 1 year after birth (Kingston et al., 2018; Shorey et al., 2018). Women experiencing perinatal depressive symptoms often report persistent sadness, hopelessness, diminished pleasure or interest in daily activities, fatigue, and physiological changes in sleep and appetite (O’Hara and Wisner, 2014). These symptoms can have lasting effects on both maternal and child well-being, making perinatal depression a significant concern at both the individual and societal level (Howard and Khalifeh, 2020). Research in this domain has attempted to explore latent trajectories of perinatal depression to identify critical periods and inform targeted support programs. However, findings have been mixed (Suarez et al., 2023). Some women experience mild or low depressive symptoms throughout the perinatal period, while others exhibit high persistent symptoms or a temporary increase of them (i.e., episodic trajectories) during pregnancy or post-partum (Vanwetswinkel et al., 2022; Zhu et al., 2024). These patterns suggest that various individual and contextual factors may play a pivotal role in shaping the onset and course of perinatal depression trajectories, highlighting the need for further research to clarify depression trends. In this longitudinal study, we explored latent trajectories of perinatal depression focusing on the influence of both individual (i.e., Sensory Processing Sensitivity) (Aron and Aron, 1997) and contextual (i.e., perceived global partner’s support) factors. By examining these variables in relation to depressive symptoms across the perinatal period, our study aims to address unresolved questions and contribute to a deeper understanding of this complex phenomenon.

Contextual factors such as socio-economic status, relationship quality, and partner support, are well known to play a significant role in women’s mental health during the perinatal period (Antoniou et al., 2022; Yang et al., 2022). Among these, partner support - including all psychological and behavioral actions offered by the partner to help women cope with the demands of the perinatal phase—is considered one of the most important sources of aid (Antoniou et al., 2022). According to meta-analytic findings, global partner support, encompassing both instrumental support (i.e., practical help and assistance with household tasks and infants care) and emotional support (i.e., the degree to which partners reflect individuals’ emotion and enhance a sense of being accepted and understood), has been found to protect women against perinatal depression (for meta-analytic findings see Pilkington et al., 2015). Additionally, information support from partners may be relevant during the transition to motherhood, as it can enhance understanding of what this potentially challenging moment entails. Yet, informational support from partners remains under explored and little is known about its association with perinatal depression (Pilkington et al., 2015).

In addition to contextual factors, individual variables, such as a history of mental disease and specific personality traits, can also increase the risk of experiencing depressive symptoms during a challenging period like the perinatal phase (Serra et al., 2023; Park et al., 2018; Badiya et al., 2020; Wikman et al., 2020; Jacques et al., 2020; Vanwetswinkel et al., 2022). Recently, the concept of Environmental Sensitivity (Pluess, 2015) has provided a framework for understanding individual differences in responding to internal feelings and surrounding experiences. Both theoretical and empirical evidence (for a review see Greven et al., 2019) suggests that individual differ in the degree to which they perceive, process, and respond to both positive and negative exposures, with around one-third of individuals showing heightened sensitivity, as captured by the biologically based trait of Sensory Processing Sensitivity (SPS) (Aron and Aron, 1997). High SPS is characterized by an in-depth processing of cognitive and emotional inputs that reflects in increased emotional reactivity and heightened sensory sensitivity. Individual with high SPS trait are more likely to get emotionally overwhelmed, with an increased risk of experiencing frequent unpleasant emotions, anxiety and depression in adulthood, especially in unsupportive environments (Booth et al., 2015; Liss et al., 2005). Specifically, the ease of excitation and the low sensory threshold, representing the negative facets of SPS, have been positively associated with psychological difficulties, such as depression, as well as a greater frequency of physical health symptoms (Bakker and Moulding, 2012; Benham, 2006; Brindle et al., 2015; Gerstenberg, 2012; Liss et al., 2008). Consequently, it is plausible that high SPS may contribute to perinatal depression, as highly sensitive women are thought to experience pregnancy-and childbirth-related feelings more intensely, making them more susceptible to emotional challenges and at greater risk of developing depressive symptoms. This increased vulnerability is likely due to the greater awareness and sensitivity to internal and external changes, which may be particularly evident in adverse or unfavorable environments. Importantly, consistent with a differential susceptibility effect (Belsky and Pluess, 2009), high sensitivity is not solely a negative disposition. It may also confer greater responsiveness to positive aspects of the environment, enabling highly sensitive individuals to benefit from enriched experiences. Thus, highly sensitive women would not necessarily be meant to get overwhelmed by the perinatal challenges but may instead thrive in supportive contexts.

Despite its relevance, the role of heightened sensitivity in perinatal depression remains unexplored. Moreover, most existing research has explored depression trajectories and related variables separately, highlighting the need for further studies to investigate the role of individual and contextual variables across different time points in the perinatal period. Understanding how (and if) depression changes during the perinatal, along with identifying potential risk and protective factors in these trajectories, is essential for providing targeted support to mothers, particularly those high in sensitivity, during more challenging periods.

The investigation of maternal perinatal depression is also crucial as its effects extend beyond the individual, significantly impacting offspring’s concurrent and long-term development (Saharoy et al., 2023).

Importantly, maternal depression experienced during pregnancy appears to affect children’s early emotional adjustment. Consistent with the prenatal programming hypothesis - which posits that prenatal stress enhances developmental plasticity, making children more permeable to both adverse and supportive postpartum experiences (Hartman and Belsky, 2018) - prenatal depressive symptoms have been linked to stronger emotional reactions and emotional regulation difficulties in infants, as captured by the negative affect trait. Temperamentally, infants with high negative affect show fussy behaviors, low adaptability and regularity, and heightened reactivity to stimuli, which may predispose them to later internalizing and externalizing problems (Chess et al., 1963; Eisenberg et al., 2009; Nigg, 2006). Research into factors influencing infants’ negative emotionality has gained increasing interest, even given the moderating role negative emotionality plays in the relationship between rearing environment and emotional adjustment (Kochanska et al., 2007). However, findings on the relationship between prenatal depression and infants’ temperament remain mixed (see Erickson et al., 2017; Sörensen et al., 2024 for reviews) or have focused only on the broad negative affect dimension without exploring specific temperamental facets of negative affect, such as fear, sadness, and anger/frustration (Spry et al., 2020).

The present longitudinal study investigated depression trajectories over time in potentially typical perinatal situations. A sample of 88 women recruited from the general population (i.e., a non-clinical sample) was followed from pregnancy to 1 year postpartum. We addressed three aims. First, we explored the latent trajectories of depression across four time points during the perinatal period: the last trimester of pregnancy, and three, six-and nine-months post-partum. Given the controversial findings in the literature, our investigation was primarily exploratory in nature. Second, we explored the role of individual trait of SPS and perceived partner support on depression across the four different time points considered. Drawing on literature on sensitivity, we anticipated high SPS predicting stronger depression symptoms, especially when partner support was low, with partner support acting as a protective factor.

Finally, we explored the association between prenatal depressive symptoms and infants’ negative affect at 3 months of age, as an early marker of individual emotional adjustment that could influence the development in the long run. We expected that greater maternal depression symptoms during the prenatal period would predict higher levels of infants’ negative affect.

## Method

### Participants and procedure

The current sample was part of a broader observational longitudinal study conducted in Central Italy and involving approximately 130 mothers. For this study, we relied on 88 mothers who completed measures of variables of interest. Mothers were asked to complete self-report questionnaires at four assessment waves: during the last trimester of pregnancy and at 3, 6, and 9 months postpartum. Mothers had a mean age of 35.03 years (*SD* = 4.92; age range: 22–43 years) and an average of 16.48 education years (*SD* = 2.87; age range: 8–21 years), indicating high level of education (i.e., bachelor’s degree or higher). Additionally, 97% of the mothers were Italian, 96% were married or in a stable relationship, 48% had a medium to high monthly income, an 81% were employed in stable jobs.

Recruitment primarily occurred during prenatal classes, where mothers were invited to participate. To reach a sample as large as possible based on available resources (see Lakens, 2022) additional recruitment was carried out in neonatal units and through the distribution of flyers. During pregnancy and at 3, 6, and 9 months of infant life, mothers were asked to fill out an online survey self-reporting on depression and perceived partner support. During pregnancy, they also reported on their sensory processing sensitivity, considered a stable trait. When infants were 3 months old, mothers reported on their infants’ temperament. At the three-month wave, the infants had an average age of 3.07 (*SD* = 0.26; age range: 3–4 months). All infants were Italian, 50% were boys, and 78% were first-born. Mothers were compensated with 15 euros for their participation in each session. Informed consent was obtained from all participants, who were fully informed about the study conditions. The study adhered to the ethical standards outlined by the American Psychological Association and by the academic Italian Association of Psychology. It was approved by the Departmental Ethics Review Board of the University of G. d’Annunzio, Chieti-Pescara, Italy.

### Measures

#### Maternal depression

Maternal depression was measured at each time point using the Edinburgh Postnatal Depression Scale (EPDS) (Cox et al., 2014; Benvenuti et al., 1999), one of the most widely used screening tool for perinatal depression. The EPDS is a 10-item self-report scale rated on a 4-point Likert scale ranging from 0 = *Absence of symptoms* to 3 = *High severity of symptoms*, with higher scores indicating more intense depressive symptoms. It evaluates a broad range of depressive symptoms experienced during the previous week, including depressed mood (e.g., “I have been so unhappy that I have been crying”), feelings of guilt (e.g., “I have blamed myself unnecessarily when things went wrong”), anxiety (e.g., “I have been anxious or worried for no good reason”), sleep disturbances (e.g., “I have been so unhappy that I have had difficulty sleeping”), and thoughts of self-harm (e.g., “the thought of harming myself has occurred to me”).

In the current study, Cronbach’s *α* values were 0.84, 0.85, 0.81, and 0.83 for pregnancy, 3, 6, and 9 months postpartum, respectively, indicating a satisfactory internal consistency.

#### Sensory Processing Sensitivity

Sensory Processing Sensitivity (SPS) is considered as a stable individual trait and was assessed only once, during pregnancy, using the Highly Sensitive Person Scale (Pluess et al., 2023; Lionetti et al., 2024). The 12-item scale is rated on a 7-point Likert scale ranging from 1 = *Not at all* to 7 = *Extremely* and captures an increased appreciation of, and a greater awareness of subtleties (e.g., “Do you seem to be aware of subtleties in your environment?”), a strong feeling of getting overwhelmed, and a low sensory threshold (e.g., “Do changes in your life shake you up?”; “Are you easily overwhelmed by things like bright lights, strong smells, coarse fabrics, or sirens close by?”). Higher scores indicate higher levels of sensitivity trait.

In the current study, Cronbach’s *α* was 0.74 indicating a satisfying internal consistency of the scale, comparable to that reported in previous studies with different samples (Pluess et al., 2023; Lionetti et al., 2024; Sperati et al., 2024b).

#### Perceived partner support during pregnancy

Perceived partner support during pregnancy was measured was assessed using the self-report Maternal Support Scale (Lutz et al., 2012), which examines specific types of family support. We specifically adopted from this scale items related to the support provided by the partner. The seven items allow to simultaneously capture emotional, instrumental, and informational support self-perceived by women from her partner. Mothers were asked to indicate the presence or absence of specific types of support from their partner using a dichotomous scale (i.e., 0 = *Absent*, 1 = *Present*). The list included emotional support (e.g., sharing of the pregnancy experience, assistance in resting when needed), informational support (e.g., assistance with obtaining information), instrumental support (e.g., cleaning, cooking). We considered a global support score, with higher scores indicate greater partner support.

#### Perceived partner support during the post-partum

Perceived partner support at 3, 6, and 9 months was assessed using the self-report Maternal Support Scale (Lutz et al., 2012), which examines specific types of family support. We specifically adopted from this scale items related to the support provided by the partner. The seven items allow to simultaneously capture emotional, instrumental, and informational support self-perceived by women from her partner. Mothers rated their perception on a 4-point Likert Scale ranging from 0 = *Not at all* to 3 = *A lot*. The questionnaire assessed various type of support provided by their partner, including emotional support (e.g., dedication from the partner, assistance in resting when needed), informational support (e.g., assistance with obtaining information), and instrumental support in household tasks (e.g., cleaning, cooking) and in baby care (e.g., changing diapers). We considered a global support score, with higher scores indicate greater partner support.

Cronbach’s *α* indicated a satisfactory internal consistency of the scale, with values of 0.81, 0.87, and 0.89 for 3, 6, and 9 months, respectively.

#### Infant negative affect

Infant’s negative affect was measured at 3 months using the Infant Behavior Questionnaire-Revised (IBQ-R) (Gartstein and Rothbart, 2003). Mothers rated the frequency of various infant behaviors over the previous week on a 7-point Likert scale ranging from 1 = *Never* to 7 = *Always*. The negative affect total score was calculated as the mean of scores across three subscales: frustration/distress to limitations (e.g., “When placed on his/her back, how often did the baby fuss or protest?”), fear (e.g., “How often during the last week did the baby startle to a sudden or loud noise?”) and sadness (e.g., “Did the baby seem sad when the caregiver was gone for an unusually long period of time?”). Higher scores indicate higher negative affect.

In the current study, Cronbach’s *α* indicated an acceptable to good internal consistency, with values of 0.82, 0.64, 0.80, 0.72 for the negative affect total score, frustration/distress to limitations, fear, and sadness, respectively.

### Data analysis

We first explored percentage of missing values for each subject across the four time points and we included participants who had data for at least three out of the four waves (i.e., only 25% of missing per subject).

We then performed a series of latent growth analyses to explore trajectories of depression across the four perinatal time points considered (i.e., during pregnancy, and at three, six, and nine month post-partum). We estimated the intercept (i.e., initial mean levels) and slope (i.e., rates of change over time) of depression as latent variables, modelling the slope as both a linear and a quadratic curve. To handle with missing data (i.e., 7% in the final sample), we adopted the maximum likelihood estimation that allow approximating unbiased parameters in the latent growth model by utilizing all available data (Enders, 2010). Finally, we plotted the estimates to provide a follow-up graphical exploration of latent trajectories of maternal depression over time.

As for the second and third aims, we included in the analyses mothers’ SPS measured at pregnancy as a stable trait, perceived support from partners assessed across the four waves, and infants’ negative affect temperament dimension with its subscales (i.e., fear, sadness, and frustration). After including the new variables of interest, we first explored the missing values for each subject across the four time points including participants with up to 30% of missing (i.e., at most one missing value per variable across the four time points). We then computed descriptive statistics and bivariate associations among all study variables at each time point. We considered associations to be low when Pearson’s *r* was around 0.10 or less, medium if *r* varied around 0.30, and large if *r* was higher than 0.50 (Cohen, 1988, 1992).

Then, we run a series of multivariate regression models. Specifically, to explore the effect of individual levels of SPS, perceived support from partners and their interaction on depressive symptoms within each perinatal time points (i.e., second aim), we estimated both the main effect (i.e., SPS + perceived support) and the interaction effect (i.e., SPS * perceived support) with sensitivity trait and partner support measured at each wave in predicting maternal depression at the four considered time points. Maximum likelihood estimation was used to handle missing data (i.e., 10% in the final sample) (Enders, 2010).

Finally, we run a multivariate regression model with mothers’ depression during pregnancy in predicting infants’ negative affect temperament dimension. Specifically, we examined both the broad temperament dimension of negative emotionality and specific subscales, such as sadness, fear, and frustration to limitations, to determine whether maternal depression during pregnancy was associated with specific negative reactivity facets potentially linked to internalizing and externalizing problems at older ages. Maximum likelihood estimation was used to handle missing data (i.e., 5% in the final sample) (Enders, 2010). For exploratory purposes, we run a series of t-test for independent sample to compare means of examined variables between excluded and included participants. Data were analyzed using R, version 4.0.0 (R Core Team, 2020).

## Results

### Descriptive statistics and bivariate associations

Descriptive statistics and bivariate associations among all study variables are reported in Table 1. Overall, results showed that depression levels were significantly associated across waves (*r* ranged from 0.55 to 0.71). Mothers’ SPS levels were moderately and positively associated with depression across all four perinatal stages (*r* ranged from 0.36 to 0.49), whereas trivial associations were found between SPS and perceived partner support during the perinatal period (*r* ranged from 0.11 to 0.08). Maternal depression during pregnancy was positively and moderately associated with mother-reported infants’ negative affect at 3 months (*r* = 0.36), with this association mainly driven by the fear temperament subscale (*r* = 0.39). Mothers’ age and income were negatively and moderately associated with depression at each wave.

**Table 1:** Bivariate associations among all study variables (*N* = 88).

	M(SD; range)	1	2	3	4	5	6	7	8	9	10	11	12	13	14
1 SPS	4.47(0.81; 3.00–6.25)														
2 EPDS_preg	0.74(0.46; 0.00–1.90)	0.34													
3 EPDS_3	0.80(0.47; 0.00–2.50)	0.42	0.63												
4 EPDS_6	0.66(0.40; 0.00–1.70)	0.49	0.71	0.66											
5 EPDS_9	0.62(0.43; 0.00–1.90)	0.40	0.71	0.55	0.67										
6 Supp_preg	4.04 (1.41; 0.00–6.00)	−0.10	−0.36	−0.21	−0.39	−0.36									
7 Supp_3	2.42(0.48; 0.86–3.00)	0.03	−0.13	−0.19	−0.32	−0.41	0.48								
8 Supp_6	2.45(0.58; 0.28–3.00)	0.06	−0.20	−0.38	−0.38	−0.29	0.54	0.73							
9 Supp_9	2.32(0.66; 0.00–3.00)	0.08	−0.20	−0.20	−0.36	−0.30	0.53	0.75	0.85						
10 NegAff	3.35(0.58; 1.80–4.60)	0.17	0.36	0.31	0.36	0.46	−0.14	−0.21	−0.09	0.04					
11 Fear	1.81(0.97; 0.17–6.00)	0.13	0.39	0.23	0.34	0.41	−0.11	−0.12	−0.02	0.06	0.69				
12 Sad	3.20 (1.15; 1.17–6.17)	0.06	0.17	0.21	0.25	0.15	0.04	−0.17	0.00	0.05	0.73	0.29			
13 Frust	3.51 (1.00; 1.57–5.57)	0.12	0.14	0.32	0.29	0.38	−0.04	−0.14	−0.08	−0.03	0.73	0.29	0.62		
14 Maternal Age		0.07	−0.31	−0.17	−0.33	−0.35	0.28	0.13	0.13	0.29	0.02	−0.08	−0.04	0.04	
15 SES		0.01	−0.19	−0.30	−0.34	−0.19	0.09	0.27	0.21	0.21	−0.04	−0.06	−0.05	−0.04	0.14

SPS, Sensory Processing Sensitivity; EPDS_preg, depressive symptoms at pregnancy; EPDS_3, depressive symptoms at 3 months; EPDS_6, depressive symptoms at 6 months; EPDS_9, depressive symptoms at 9 months; Supp_preg, perceived partner support during pregnancy; Supp_3, perceived partner support at 3 months; Supp_6, perceived partner support at 6 months; Supp_9, perceived partner supp at 9 months.

### Maternal perinatal depression trajectories

**Figure 1:**
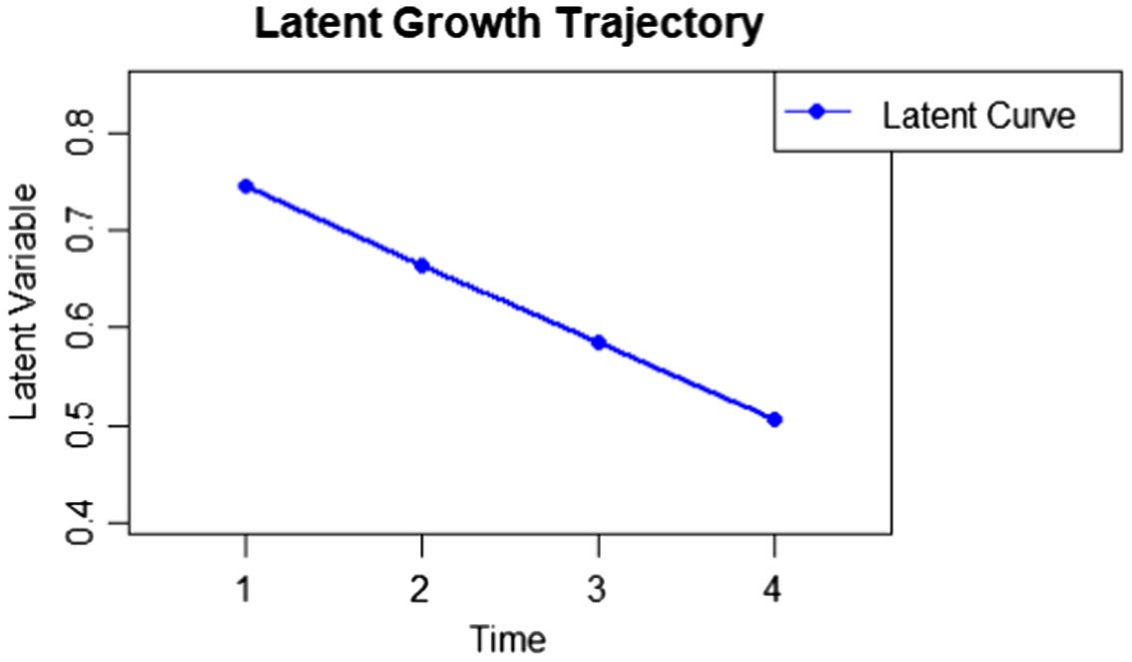
Graphical exploration of the latent trajectory of maternal depression across the perinatal waves.

According to a preliminary exploration of missing data, *n* = 17 subjects had more than one missing per wave, that is more than 25% of missing data across time points and were thus excluded. The excluded subjects did not differ from the included ones on mean of depression at each time. Results from the latent growth analysis showed that maternal depression in our sample had an initial average level of 0.83 (intercept, *β* = 0.83(0.06), *p* < 0.001), which is lower than the cut-off of 1.3 identified for the non-clinical mothers in the validation study (Benvenuti et al., 1999). A significant linear decrease in depression scores over time was identified (linear slope, *β* = −0.08(0.02), *p* < 0.001). Variance in the intercept was significant (*β* = 0.17, SE = 0.05, *p* < 0.01) but variance over time was not significant (*β* = 0.001, SE = 0.005, *p* = 0.85). When a quadratic slope was included, the estimated parameters for the quadratic slope was non-significant (quadratic slope, *β* = 0.02(02), *p* = 0.28). To explore graphically the latent trajectories of maternal depressive symptoms, we plotted trends (see Figure 1). The follow-up plot showed an overall decreasing pattern across the four time points.

### The role of SPS and partner support in predicting perinatal depression symptoms

According to missing exploration, we excluded *n* = 28 subjects who had more than 30% of missing data across time points. The excluded subjects did not differ from the included ones on any examined variable at each time point, except for support during pregnancy. For this variable, the mean perceived support during pregnancy was significantly lower in the excluded group compared to the included group (*p* < 0.001). Furthermore, a preliminary exploration showed that regression assumptions were met and residuals were approximately normally distributed.

Results from the multivariate regression models are reported in Table 2. The estimated parameters suggested a significant main effect of SPS in predicting greater perinatal depressive symptoms across all four waves. In other words, mothers higher in SPS levels were those who reported more intense depressive symptoms during the perinatal period. Regarding perceived support from partner, results showed that support during pregnancy was negatively and significantly associated with depression experienced during pregnancy (*β* = −0.42, *p* = <0.01). A significant effect of support during pregnancy was also found in predicting lower depression at 6 months postpartum (*β* = −0.32, *p* = 0.03), suggesting a potential long-term effect pattern alongside the concurrent one. Regarding perceived partner support at 3 and 9 months postpartum, results suggested a non-significant effect in predicting depressive symptoms.

**Table 2:** Estimated parameters of multivariate regression models with sensory processing sensitivity and partner support at pregnancy, 3, 6, and 9 months predicting maternal depressive symptoms the perinatal timepoints (*N* = 60).

	EPDS_preg			EPDS_3			EPDS_6			EPDS_9		
	ß	*p*	R square	ß	*p*	R square	ß	*p*	R square	ß	*P*	R square
SPS	**0.29**	**0.021**	0.29	**0.41**	**<0.01**	0.23	**0.45**	**<0.001**	0.36	**0.33**	**0.018**	0.31
Support_preg	**−0.42**	**0.001**		−0.16	0.347		**−0.32**	**0.030**		−0.31	0.061	
Support_3				−0.07	0.599		−0.16	0.294		−0.33	0.097	
Support_6							0.17	0.240		0.09	0.632	
Support_9										0.15	0.479	
SPS * Supp preg	1.04	0.11										
SPS * Support_3				−0.89	0.542							
SPS * Support_6							−0.18	0.919				
SPS * Support_9										2.405	0.088	

SPS, Sensory Processing Sensitivity; EPDS_preg, depressive symptoms at pregnancy; EPDS_3, depressive symptoms at 3 months; EPDS_6, depressive symptoms at 6 months; EPDS_9, depressive symptoms at 9 months; Support_preg, perceived partner support during pregnancy; Support_3, perceived partner support at 3 months; Support_6, perceived partner support at 6 months; Support_9, perceived partner support at 9 months. Significant regressions are marked in bold.

When the interaction terms were added to the main effects, results showed no evidence for any interactions between SPS and perceived support from partner.

### The role of maternal depression in pregnancy in predicting infants’ negative affect

According to missing exploration, we excluded *n* = 17 subjects who had more than 20% of missing data across two time points. The excluded subjects did not differ from the included ones on any variable of interest.

When estimating the multivariate regression model, results showed that depression during pregnancy significantly predicted negative affect (*β* = 0.34, *p* = 0.029), with prenatal depression explaining 12% of the variance in infants’ negative affect. Specifically, when subscales were considered, the effect appeared to be driven by the fear subscale (*β* = 0.46, *p* = 0.001), which accounted for 22% of the variance in fear. In contrast, sadness (*β* = 0.17, *p = 0.*32) and frustration (*β* = 0.10, *p* = 0.586) were not significantly predicted by maternal depression, explaining only 3 and 1% of their variance, respectively.

## Discussion

This longitudinal study explored how maternal depression changes during the perinatal period, focusing on the role of an underexplored individual factor (i.e., the Sensory Processing Sensitivity trait) and a contextual factor (i.e., partner support) in shaping depressive experience. It also investigated the impact of prenatal depression on infants’ early emotional adjustment.

The exploration of the latent trajectories of depression suggests an overall decrease in symptoms over time, consistent with expectation for a non-clinical sample (Benvenuti et al., 1999). Interestingly, both observed means and graphical exploration of latent estimates indicate that the period surrounding childbirth seems to be critical, during which mothers appear more susceptible to mood declines. This finding aligns with previous studies suggesting higher rates of women’s depression most commonly within the 6 weeks post-partum in non-clinical samples (Gavin et al., 2005; Woody et al., 2017). However, these elevated depressive symptoms do not reach clinical thresholds when compared to the cut-off score, and the initial mean was comparable to findings from the Italian validation study with non-clinical mothers (Benvenuti et al., 1999). Moreover, depression levels in our sample remained overall low throughout the perinatal period, as also indicated by the small effect size observed for the negative linear trend. This suggests that the slightly elevated negative feelings identified between late pregnancy and 3 months post-partum may reflect hormonal fluctuations and birth-related physiological processes, making a mild degree of depression mood a potentially typical and expected phenomenon (Antoniou et al., 2022).

When considering the effect of individual trait of SPS on depression levels at each perinatal time point, high SPS emerged as a potential risk factor. Specifically, women higher in SPS were more likely to experience pronounced mood declines than less-sensitive ones, particularly in the post-partum period (i.e., at 3 and 6 months). This suggests that the transition to motherhood may be particularly challenging for highly sensitive mothers, who might need more resources and psychological support to adapt effectively. This is likely because highly sensitive mothers, due to their deeper processing of internal and external stimuli, may be more aware of both physiological and psychological changes, including childbirth-related feelings and thoughts, leading to a greater sense of overwhelm and mood decline. This finding aligns with previous research (Booth et al., 2015; Liss et al., 2005; Sperati et al., 2024a,b) and meta-analytic evidence (Lionetti et al., 2019) linking high sensitivity to internalizing symptoms in adulthood, including negative affect, depression, stress, and anxiety. Importantly, this study extends prior research a step further, by highlighting how individual differences in sensitivity shape depressive mood during the perinatal period. These findings provide valuable insights into maternal care by emphasizing the importance of considering individual differences in how mothers respond to the challenges of becoming a parent. Recognizing that each mother may experience and adapt to this transition in unique ways highlights the critical need for a more personalized approach to support. Thus, from a more applied perspective, these insights underscore the importance of comprehensive screenings during the perinatal period in order to identify more at-risk mothers and to develop tailored parenting support programs that consider differences in individual characteristics, such as SPS. By integrating a deeper understanding of these variations, such programs can provide mothers with valuable knowledge about what it means to be highly sensitive. This awareness can empower them to better understand their own emotional responses and thoughts, to equip them for coping with stressors more effectively, and develop strategies to navigate parenthood in a way that aligns with their unique characteristics.

Although we found that high SPS increased the risk of depression at each perinatal time point, in line with the differential susceptibility effect (Belsky and Pluess, 2009) we also expected highly sensitive mothers to benefit more from supportive experiences, showing lower depression when partner support was high. However, we found no evidence of an interaction effect between SPS and partner support, likely because the main effect of SPS is relatively strong. This suggests that being a highly sensitive woman makes the perinatal period particularly challenging and that additional forms of support may be needed beyond what is typically provided by the partner. For instance, receiving emotional support and practical help from close ones, participating in peer groups for mutual self-support, as well as engaging in mindfulness-related practices could be of benefit for highly sensitive women in increasing a non-reactive attitude and reducing depressive feelings during the perinatal experience (Passaquindici et al., 2024; Passaquindici et al., under review). Indeed, these interventions could equip mothers with effective coping strategies and practical tools that could be integrated into daily routines to deal with the challenges of parenting (e.g., deep breathing to stay calm during stressful moments). Alternatively, the support provided by partners may be more relevant at specific stages of the perinatal period that we did not account for (e.g., around the time of birth) or when challenging life events occur during the postpartum period (e.g., moving, career changes), playing a particularly protective role for mothers with high sensitivity. Future research is needed to further investigate the interaction between sensitivity and partner support as a potential protective factor against maternal depression.

Regarding the role of contextual variables in perinatal depression, high perceived partner support during pregnancy has been found to predict lower maternal depression both during pregnancy and from 6 months postpartum. This suggests a concurrent and long-term buffering effect of prenatal partner support against maternal depression that is even stronger than support experienced during the post-partum period. The finding that prenatal global partner support, including both emotional, informational, and instrumental help, protects against depression during both pregnancy and after childbirth aligns previous meta-analytic findings indicating the importance of supportive partner relationships against mental health issues in women during the perinatal phase (see Pilkington et al., 2015 for meta-analytic findings). This highlight the relevance of encouraging and facilitating active participation by partners, especially during pregnancy, which can significantly enhance maternal well-being by fostering a sense of shared responsibility and emotional support, both of which are critical during this potentially challenging period. Given its significant impact, partner participation should be a key component of parenting programs designed to nurture positive relationships from the very beginning of pregnancy, laying the foundation for a supportive family dynamic and a healthier transition to parenthood.

Interestingly, our longitudinal study further clarified the role of partner support at different stages of the perinatal period. While partner support during pregnancy was associated with lower depressive symptoms during pregnancy and at 6 months (with a smaller effect at 9 months), it was not linked to depression at 3 months post-partum. This finding reinforces the idea that the period immediately following childbirth may represent a time of adjustment from both a physiological and psychological perspectives, during which some depressive symptoms commonly emerge. Thus, partner support, whether prenatal or concurrent (i.e., reported at 3 months), does not appear to significantly buffer against the typical mood decline during this period. Moreover, regarding postpartum support later in the postpartum period (i.e., at 6 and 9 months), we found no significant effects on maternal depression, as also indicated by the small effect size. This finding is likely because other types of support may play a role when different developmental tasks arise. Specifically, from 6 months postpartum, changes in family needs and routines occur, for instance, related to child development (e.g., baby weaning, daycare enrolment) or due to maternal reengagement in work. During these specific transitions, sources of support beyond that provided strictly by the partner may contribute to maternal adjustment, including family support (e.g., from the mother’s or father’s parents to help with the child care) and community networks (e.g., relationships with other families for informational support). Future studies should consider exploring the role of other types of support to deepen our understanding in this regard.

Regarding the role of prenatal maternal depression in infants’ emotional adjustment, we found that maternal depression during pregnancy significantly predicted higher levels of negative affect in infants at 3 months of age. This finding aligns with previous studies showing that mothers with prenatal depressive symptoms often perceive their infants as more difficult (Nieto et al., 2019; Zhang et al., 2018). Importantly, our study extends this knowledge by examining different facets of infant negative affect, such as fear, sadness, and frustration. We found that the association between prenatal depression and infants’ negative affect was primarily driven by the fear subscale, rather than by sadness or frustration. The lack of association with sadness may seem surprising, given its role as an early marker of internalizing distress, while fear is commonly associated with anxiety (Hankin et al., 2016). However, symptoms of depression and anxiety frequently overlap to some extent, as the EPDS also captures anxiety-related feelings, both reflecting maternal distress. According to the prenatal programming hypothesis (Hartman and Belsky, 2018), prenatal stress enhances individual reactivity to stimuli, likely through the action of stress-related hormones such as cortisol (Galbally et al., 2021; Sörensen et al., 2024). Among temperamental traits, fear has traditionally been considered an early marker of heightened emotional reactivity (e.g., increased startle responsiveness) which may explain why maternal depression in the prenatal period is associated with an increase in infants’ temperament fear. This temperament characteristic, biased toward negative emotionality, could pose challenges in emotion regulation and increase the risk of internalizing symptoms later in childhood (Eisenberg et al., 2009; Hankin et al., 2016; Nigg, 2006), especially in unfavorable rearing environments. At the same time, moving beyond the dual-risk perspective, evidence suggests a differential susceptibility effect (Kochanska et al., 2007), with more emotionally negative infants being more adversely impacted by poor mother–child relationships but also benefit more from high-quality caregiving (Pluess and Belsky, 2009). Therefore, understanding the antecedents of negative affect, and specifically the fear facet, is crucial for early identification of infants who may be more responsive to environmental influences. This knowledge can inform the development of targeted support programs aimed at fostering optimal caregiving environments based on infants’ individual differences. Finally, maternal depression has been found to be physiologically linked to mother–child interaction quality, with depressed mothers more likely to be withdrawn from her child, particularly from the tactile experience. This detachment significantly impairs the ability to provide “body-to-body” communication through affectionate touch, which in turn reduces the infant’s perceptual sensitivity to it (Nance et al., 2024) and may increased infants’ negative affect (Egmose et al., 2018). However, enhancing and promoting affective interactive touch among dyads in the post-partum period could improve mother-baby bonding, reducing stress, with benefits for both maternal emotional state and infants’ emotional adjustment (La Rosa et al., 2024). This positive role of tactile experience could inform intervention programs aimed at promoting secure and supportive relationships early in life, including among dyads suffering from some emotional difficulties.

### Strengths and limitations

Our longitudinal study presents several strengths. First, this is the first to explore perinatal depressive symptoms trajectories over four assessments waves, from the last trimester of pregnancy to 9 months postpartum. In doing so, we used a latent growth analysis, as this approach offers a more precise and reliable understanding of how depression changes unfold. Second, we investigated the role of partner support as well as the effect of an underexplored individual variable such as SPS, providing new theoretical and applied insights in the field. Finally, while previous studies have primarily examined perinatal depressive trajectories in clinical samples, our study focused on a non-clinical population, providing valuable insights into the typical physiological progression of these symptoms over time.

However, findings should be also considered in light of some limitations. First, we acknowledge that our sample might be underpowered, highlighting the need for further evidence based on larger sample sizes. Future research should replicate these findings and consider conducting an *a priori* power analysis to more accurately determine the sample size needed to detect changes in depression over the perinatal period. Second, our findings rely on parent-reported measures. While this approach is commonly used in studies with infants, further observational assessments of infant temperament and maternal mood may provide a more accurate and nuanced understanding. Second, the non-clinical sample may underestimate the impact of sensitivity and perceived partner support. Future studies should investigate whether their effects are potentially more pronounced in clinical populations. Finally, we used a non-validated questionnaire to assess perceived partner support, relying solely on a total score. It is possible that certain dimensions of partner support (e.g., emotional support) may be particularly relevant, especially for highly sensitive mothers. Future research should focus on longitudinal observational designs with clinical populations and explore facets of partner support separately to better understand their contribution in addressing perinatal depressive symptoms.

## Conclusion

The current longitudinal study advances research on maternal perinatal depression by clarifying that mood decline commonly occurs immediately following childbirth in non-clinical populations, along with a decrease in depression symptoms over the 9 months post-partum. Our findings also contribute to a more comprehensive understanding of how individual and contextual factors contribute to depression during the perinatal period. Specifically, our study suggests that high sensitivity may increase the risk of experiencing depressive mood, due to the greater awareness of both internal feelings and external birth-related stressors.

Moreover, we found that partner support experienced during pregnancy, but not postnatally, has a protective effect on both concurrent and postpartum depression from 6 months onward, except for the physiological mood decline immediately after birth.

Finally, our study suggests that prenatal maternal depression is significantly associated with infant negative affect, particularly the fear subscale, likely acting as a prenatal stressor that enhances individual reactivity to stimuli, as captured by fear temperamental marker.

Overall, these findings have the potential to inform parenting programs aimed at promoting supportive relationship during pregnancy and fostering specific interventions (e.g., peer emotional support or mindfulness-related practices) targeting all mothers, but that can be of benefit for highly sensitive ones, who are more at risk of suffering from depressive symptoms in the perinatal period with relevant effects on infants’ emotional adjustment.

## Data Availability

The raw data supporting the conclusions of this article will be made available by the authors, without undue reservation.
